# Reversal of Vitamin K Antagonist (VKA) effect in patients with severe bleeding: a French multicenter observational study (Optiplex) assessing the use of Prothrombin Complex Concentrate (PCC) in current clinical practice

**DOI:** 10.1186/cc11669

**Published:** 2012-10-04

**Authors:** Thibaut Desmettre, Emilie Dehours, Charles-Marc Samama, Suchin Jhundoo, Frédéric Pujeau, Christian Guillaudin, Claudine Hecquart, Pierre Clerson, Jean Charles Crave, Roland Jaussaud

**Affiliations:** 1 Urgences/SAMU 25, CHU Besançon, Université de Franche Comté, 1 Bd Fleming, Besançon, 25000, France; 2 Pôle de Médecine d'Urgence, CHU Purpan, Place du Dr Baylac, Toulouse, 31059, France; 3 Anesthésie-Réanimation, CHU Hôtel-Dieu, Université Paris Descartes, 1 place du Parvis Notre Dame, Paris, 75181, France; 4 Urgences, CH Saint Jean, 20 avenue du Languedoc, Perpignan, 66000, France; 5 Urgences, CH Pau, 4 Bd Hauterive, Pau, 64046, France; 6 Pharmacie, CH Saint Esprit, lieu dit saint esprit, Agen, 47000, France; 7 Pharmacie, CHU Côte de Nacre, avenue de la Côte de Nacre, Caen, 14000, France; 8 Orgamétrie biostatistiques, 84 Bd Général Leclerc, Roubaix, 59100, France; 9 Département médical, Octapharma-France SAS, 62 avenue André Morizet, Boulogne Billancourt, 92100, France; 10 Médecine Interne, Hôpital Robert Debré, Avenue du Général Koenig, Reims, 51092, France

## Abstract

**Introduction:**

Prothrombin Complex Concentrate (PCC) is a key treatment in the management of bleeding related to Vitamin K antagonists (VKA). This study aimed to evaluate prospectively PCC use in patients with VKA-related bleeding in view of the French guidelines published in 2008.

**Methods:**

All consecutive patients with VKA-related bleeding treated with a 4-factor PCC (Octaplex^®^) were selected in 33 French hospitals. Collected data included demographics, site and severity of bleeding, modalities of PCC administration, International Normalized Ratio (INR) values before and after PCC administration, outcomes and survival rate 15 days after infusion.

**Results:**

Of 825 patients who received PCC between August 2008 and December 2010, 646 had severe bleeding. The main haemorrhage sites were intracranial (43.7%) and abdominal (24.3%). Mean INR before PCC was 4.4 ± 1.9; INR was unavailable in 12.5% of patients. The proportions of patients who received a PCC dose according to guidelines were 15.8% in patients with initial INR 2-2.5, 41.5% in patients with INR 2.5-3, 40.8% in patients with INR 3-3.5, 26.9% in patients with INR > 3.5, and 63.5% of patients with unknown INR. Vitamin K was administered in 84.7% of patients. The infused dose of PCC did not vary with initial INR; the mean dose was 25.3 ± 9.8 IU/Kg. Rates of controlled bleeding and target INR achievement were similar, regardless of whether or not patients were receiving PCC doses as per the guidelines. No differences in INR after PCC treatment were observed, regardless of whether or not vitamin K was administered. INR was first monitored after a mean time frame of 4.5 ± 5.6 hours post PCC. The overall survival rate at 15 days after PCC infusion was 75.4% (65.1% in patients with intracranial haemorrhage). A better prognosis was observed in patients reaching the target INR.

**Conclusions:**

Severe bleeding related to VKA needs to be better managed, particularly regarding the PCC infused dose, INR monitoring and administration of vitamin K. A dose of 25 IU/kg PCC appears to be efficacious in achieving a target INR of 1.5. Further studies are required to assess whether adjusting PCC dose and/or better management of INR would improve outcomes.

## Introduction

Vitamin K antagonists (VKAs) are oral anticoagulants that inhibit liver production of vitamin K-dependent coagulation factors, such as factors II, VII, IX, and X and proteins C and S. Bleeding in patients treated with VKA is the most serious iatrogenic complication, leading to more than 17,000 hospitalizations per year in France (that is, 12% of hospitalizations related to adverse events) [1]. Moreover, VKA causes 5,000 deaths per year [2-6]. The incidence of bleeding associated with oral anticoagulants is expected to increase over time as the population ages.

Intracranial hemorrhage is the principal type of bleeding related to oral anticoagulants, accounting for approximately 15% of all intracranial hemorrhages [7-9]. The rates of intracranial hemorrhages range from 0.25% to 1.1% per year to about 2% when the international normalized ratio (INR) exceeds 2 and rise dramatically thereafter [10-14]. Concomitant use of antithrombotic treatments doubles the risk of intracranial hemorrhage [10]. Intracranial hemorrhages related to VKA have a high mortality rate, approaching 50% at 1 month [7]. The prognosis of patients with VKA-related intracranial hemorrhage is generally poor compared with that of patients with spontaneous intracranial hemorrhage. In these patients, the hemorrhage is larger at baseline and hematoma expansion may persist after admission [15-17]. Hematoma size is a major predictor of mortality and worsening neurological condition. Early and rapid INR correction is therefore crucial in the management of these patients [18]. Coagulation reversal should be initiated as soon as symptom onset occurs to prevent hematoma expansion [16,19-21].

In July 2008, the French National Health Authority (Haute Autorité de Santé) published guidelines for managing patients with bleeding complications related to oral anticoagulants by elective or emergency surgery or other invasive procedures [1]. In these guidelines, prothrombin complex concentrate (PCC) was recommended for rapid INR normalization (INR of less than 1.5) in patients with VKA-related bleeding.

Previous published data showed that the management of VKA-related intracranial hemorrhage was not in line with current recommendations in European countries [22]. In France, PCC remains under-used in the treatment of severe hemorrhage and physicians do not always follow the recommended dosage [23]. Efforts thus should be made to follow recommendations in the choice of indications, dosage, and coagulation monitoring.

Octaplex^® ^(Octapharma, Lachen, Swizerland) is a human plasma-derived four-factor PCC, including factors II, VII, IX, and X, and has undergone detergent treatment and nanofiltration for viral inactivation. This product also contains proteins C and S, two natural factors limiting the extension of the coagulation process [24].

We conducted a prospective observational study (Optiplex study) between 2008 and 2010 to describe the current use of PCC. The main objective was to assess the current management of patients with severe bleeding associated with VKA and treated with PCC.

## Materials and methods

### Patients

Optiplex was a multicenter prospective observational study conducted in 33 French hospitals between August 2008 and December 2010. Patients were given the usual care to manage bleeding related to VKA. Ethical approval therefore was not sought, and informed consent was not obtained. Data were collected anonymously. In each participating center, PCC was stored in the central pharmacy and delivered upon request, mainly to emergency departments or intensive care units. The patients included were identified from the list of patients prescribed PCC at each central pharmacy. Patients had to meet the following criteria to be included in the study: they had to be at least 18 years old and treated with VKA and had to have received PCC for spontaneous or traumatic hemorrhage or for the management of increased bleeding risk before unplanned surgery. Severe bleeding was defined as life-threatening or organ-compromising bleeding, external bleeding uncontrolled with conventional measures, bleeding with hemodynamic instability (systolic blood pressure of less than 90 mm Hg or systolic blood pressure decrease of at least 40 mm Hg or mean blood pressure of less than 65 mm Hg or any signs of shock), or hemorrhage requiring urgent surgery or red cell transfusion.

### Collected data

Collected data included demographics, indication for VKA therapy, and site and severity of bleeding. Concomitant coagulation disorders such as disseminated intravascular coagulation (DIC) syndrome or fibrinolysis and history of heparin-induced thrombocytopenia were reported. If transfusion was required, the type and volume of product - red cells, platelet concentrates, and fresh frozen plasma (FFP) - were reported. The Beyth score was calculated by using the Outpatient Bleeding Risk Index. One point was given for each of the following: (a) age of 65 or more, (b) history of gastrointestinal bleeding, (c) history of stroke, and (d) one or more comorbid conditions (recent myocardial infarction, anemia, renal impairment, or diabetes mellitus). The patient was at low risk if the score was 0, moderate risk if the score was 1 or 2, and high risk if the score was 3 or more. The initial INR value, which was available before infusion, was reported. A note was made whenever the initial INR value was unavailable. Details concerning PCC administration, including the time and dose of the first administration, any subsequent administration, and simultaneous administration of vitamin K, were assessed. INR values and results of blood testing within 24 hours of PCC administration were collected. Clinical outcomes such as bleeding control and INR normalization (target INR of less than 1.5) were also evaluated. Outcomes at day 15 after PCC infusion, including death, thromboembolic events, and DIC, were assessed. Data on anticoagulation treatments, VKA, or heparin in the 15 days following PCC administration were collected.

### Statistical analysis

Data were analyzed by using SAS 9.1 software (SAS Institute Inc., Cary, NC, USA). Categorical data were described by frequency and percentages; continuous data were summarized by their mean and standard deviation. Comparisons were assessed by using the analysis of variance, Student *t *test, or Wilcoxon test for continuous variables and the chi-squared or Fisher exact test for categorical variables. Time from PCC administration to death was estimated with the Kaplan-Meier method. Events were censored at 15 days after PCC infusion. Hazard ratio and two-sided 95% confidence interval were estimated from univariate and multivariate Cox models. Comparisons between strata were assessed with the log-rank test.

## Results

**Figure 1:**
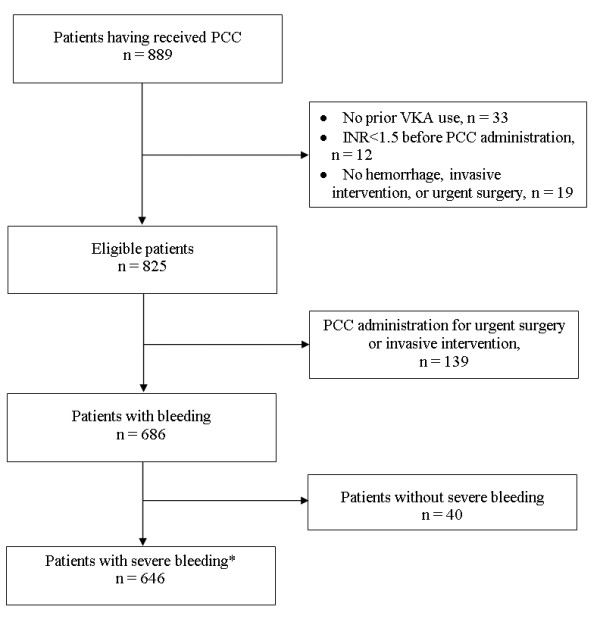
**Study flowchart**. *Severe bleeding was defined as life-threatening or organ-compromising bleeding, external bleeding uncontrolled with conventional measures, bleeding with hemodynamic instability (systolic blood pressure of less than 90 mm Hg or systolic blood pressure decrease of at least 40 mm Hg or mean blood pressure of less than 65 mm Hg or any signs of shock), or hemorrhage requiring urgent surgery or red cell transfusion. INR, international normalized ratio; PCC, prothrombin complex concentrate; VKA, vitamin K antagonist.

In total, 825 patients (that is, 89% of patients who received PCC during the study period) were included in the analysis. Patients were admitted to emergency departments or intensive care units. Of these analyzed patients, 139 received PCC for the management of bleeding risk prior to surgical or invasive intervention and 686 patients for a bleeding episode (Figure 1).

**Figure 2:**
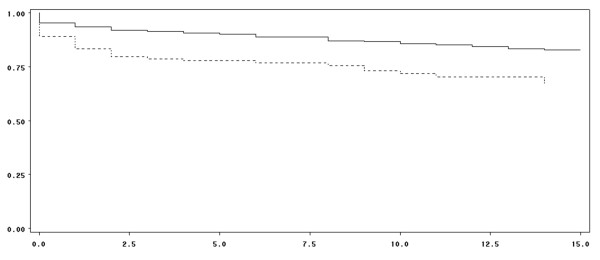
**Survival distribution function according to achievement of target international normalized ratio (INR)**. Full line: patients reaching target INR. Dotted line: patients not reaching target INR. X-axis: Days after prothrombin complex concentrate infusion. Y-axis: Survival distribution function. Estimated survival rates were 83.0% in patients reaching target INR and 65.5% in patients not reaching target INR (log-rank *P *< 0.0001).

**Figure 3:**
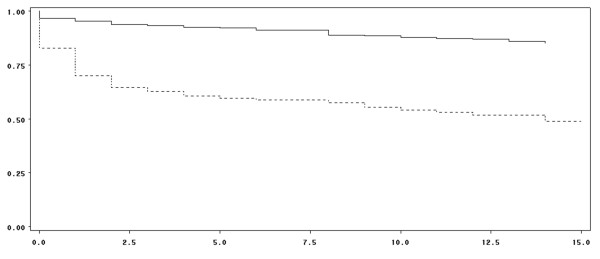
**Survival distribution function according to bleeding control**. Full line: Patients with controlled bleeding. Dotted line: Patients with uncontrolled bleeding. X-axis: Days after prothrombin complex concentrate infusion. Y-axis: Survival distribution function. Estimated survival rates were 84.5% in patients with controlled bleeding and 49.0% in patients with uncontrolled bleeding (log-rank *P *< 0.0001).

In patients treated with PCC for bleeding, the mean age was 78 years and 54% were men. Baseline characteristics of patients with bleeding and distribution of bleeding sites are shown in Tables 1 and 2, respectively. For 143 patients (22%), a concomitant anticoagulant therapy with antiplatelets was reported. According to the Beyth score [25], 59 patients (8.6%) were at high risk of bleeding. Of these, 19 (32.2%) had a history of gastrointestinal bleeding. Intracranial hemorrhages and acute gastrointestinal bleeding were the main types of severe bleeding. Gastrointestinal bleeding and hematuria were more frequent in patients treated with antiplatelet drugs (*P *= 0.041). Criteria for severe bleeding in the 646 patients are shown in Table 1. The INR was available before PCC administration in 600 patients (87.5%) regardless of the severity of bleeding. Table 3 shows the expected and infused doses of PCC according to the initial value of INR and the clinical outcomes. The mean value of INR was 4.4 ± 1.9 (median of 3.8) before PCC infusion. In patients with intracranial hemorrhage (*n *= 300), the mean INR value was 3.5 ± 1.5 (median of 3.2). The INR was considerably increased, exceeding 3.5, in 337 patients (56.2% of patients with documented INR). It was increased in the remaining patients as follows: 33 (5.5%) had an INR within 1.5 to 2, 89 (14.8%) within 2 to 2.5, 69 (11.5%) within 2.5 to 3, and 72 (12.0%) within 3 to 3.5. The mean infused PCC dose was 25.3 ± 9.7 IU/kg (range of 5.3 to 80). There was no difference between PCC infused doses with regard to the initial INR value. For 226 patients (32%), PCC was administered as a bolus dose (defined by an infusion rate of greater than 8 mL/minute). Vitamin K was administered in 576 patients (84.7%). No differences were noted between the value of INR after PCC treatment in the group that received vitamin K compared with the group that did not receive vitamin K, regardless of the time of INR measurement: H+30 minutes, H+60 minutes, H+6 hours, and H+24 hours. INR was first monitored after a mean time frame of 4.5 ± 5.5 hours after infusion and subsequently after a mean time of 11.9 ± 5.7 hours. Target INR (< 1.5) was reached in 452 patients (78.5%). In 163 patients, mean INR was 1.50 ± 0.58 one hour after PCC infusion. The proportion of patients reaching target INR was similar regardless of the initial INR value (*P *= 0.18). A comparison of the group of 'target INR achievers' versus 'INR non-achievers' showed no differences concerning age, antiplatelet treatment, location of hemorrhage, and administration of vitamin K. Bleeding was completely controlled in 458 patients (79.1% of the overall population). Bleeding control was reported in 170 patients (85.4%) receiving a lower PCC dose than expected, in 104 (77.6%) of the patients receiving the expected PCC dose, and in 50 (70.4%) of the patients receiving a higher dose than expected (*P *= 0.02). Table 4 shows the proportion of patients who received PCC doses in compliance with French recommendations. The estimated survival rate within 15 days of PCC administration was 75.4% in the overall population. No difference in survival was found between groups with or without antiplatelet drugs. However, survival was found to be significantly impacted by target INR achievement and bleeding control (*P *< 0.0001 for both) (Figures 2 and 3). Bleeding control and target INR achievement were independent predictors of survival: for bleeding control, hazard ratio (HR) was 0.28 (0.18 to 0.43) (*P *< 0.0001); for INR achievement, HR was 0.52 (0.34 to 0.81) (*P *< 0.004) (multivariate Cox model). In patients with intracranial hemorrhage, the survival rate was 65.1% at 15 days after infusion. Achievement of target INR (*P *= 0.02) and control of bleeding (*P *< 0.0001) had a significant impact on the survival rate in this population, whereas antiplatelet drugs had no impact. There was no difference in the proportion of patients reaching target INR regardless of concomitant vitamin K administration (*P *= 0.89). However, bleeding control was significantly higher in patients supplemented with vitamin K (81% versus 70% of patients not supplemented; *P *= 0.04). Moreover, respecting the recommended dose had no impact on survival (*P *= 0.16, log-rank test). In patients followed for at least 14 days after PCC infusion, VKA was resumed in 136 patients (26.4% of the overall population) and in 21 patients (10.5%) with intracranial hemorrhage (Table 5). After PCC infusion, four patients experienced thromboembolic events: deep venous thrombosis was diagnosed in three patients at six days, eight days, and 21 days, respectively, and stroke was diagnosed in one patient at two days after PCC infusion. The stroke was considered by the physician to be possibly related to PCC infusion. One patient experienced DIC due to septic shock at 21 days after PCC infusion.

**Table 1:** Characteristics of patients with bleeding (*n *= 686)

Demographic data	
Age in years, mean ± SD	77.8 ± 10.8

Men, number (percentage)	368 (53.7)

Weight in kilograms, mean ± SD	72.0 ± 16.2

VKA indication^a^, number (percentage)	

Non-valvular atrial fibrillation	449 (65.5)

Venous thromboembolic disease	131 (19.1)

Mechanical heart valve	47 (6.9)

Not available	89 (13.0)

Other anticoagulant treatment^b^, number (percentage)	

Antiplatelet drugs	143 (22)

Outpatient bleeding risk index: Beyth score, number (percentage)	

Mild risk	41 (6.0)

Intermediate risk	586 (85.4)

High risk	59 (8.6)

Criteria of severe bleedings (*n *= 646), number (percentage)	

Life-threatening bleeding or organ-compromising bleeding	574 (88.3)

Hemodynamic instability	147 (21.4)

External bleeding uncontrolled with conventional measures	167 (26.3)

Need for red cell transfusion	217 (31.6)

Need for urgent surgery	162 (23.6)

^a^More than one indication was possible; 89 values were missing; ^b^36 values were missing. Outpatient bleeding risk index was calculated according to Beyth score [29]. Hemodynamic instability was defined as the presence of one or more of the following criteria: systolic blood pressure (SBP) of less than 90 mm Hg, SBP decrease of at least 40 mm Hg, mean blood pressure of less than 65 mm Hg, or any signs of shock. SD, standard deviation; VKA, vitamin K antagonist.

**Table 2:** Description of bleeding sites (*n *= 686)

Site	Number (percentage)
Intracranial	300 (43.7)

Gastrointestinal	167 (24.3)

Deep muscle hematoma or neural compression syndrome or both	65 (9.5)

Hemoperitoneum/Hemoretroperitoneum	59 (8.6)

Hemothorax	26 (3.8)

Hematuria	22 (3.2)

Hemoptysis	17 (2.5)

Hemopericardium	6 (0.9)

Hemarthrosis	6 (0.9)

Intraocular or retro-orbital	4 (0.6)

Intraspinal	2 (0.3)

Unknown	37 (5.4)

Multiple sites of bleeding were observed in several patients: 22 patients with two sites, four patients with three sites, and one patient with four sites.

**Table 3:** Expected and infused prothombin complex concentrate dose according to initial international normalized ratio

Initial INR	2-2.5 (*n *= 57)	2.5-3 (*n *= 53)	3-3.5 (*n *= 49)	> 3.5 (*n *= 249)	Unavailable (*n *= 71)
Expected PCC dose, IU/kg					
Mean	10	20	25	> 30	25
Range	7.5-15	15-22.5	22.5-30		

Infused PCC dose, IU/kg					
Mean ± SD	24.9 ± 9.6	22.5 ± 6.9	27.1 ± 9.3	26.2 ± 10.2	25.2 ± 9.4

Target INR achievement^a^	40 (81.6)	37 (84.1)	30 (79.0)	171 (79.9)	46 (74.2)

Bleeding controlled^a^	38 (74.5)	34 (80.0)	27 (81.8)	175 (82.2)	50 (76.9)

Results are expressed as number (percentage) or as mean ± standard deviation (SD). The results are presented in patients with documented prothombin complex concentrate (PCC) dose and initial international normalized ratio (INR) of at least 2 or unavailable INR (*n *= 479). ^a^Percentages are calculated on the number of patients with a documented response.

**Table 4:** Patients receiving prothombin complex concentrate dose in compliance with recommendations of the French National Health Authority (Haute Autorité de Santé)

Initial international normalized ratio	2-2.5 (*n *= 57)	2.5-3 (*n *= 53)	3-3.5 (*n *= 49)	> 3.5 (*n *= 249)	Unknown (*n *= 71)
Patients with infused dose < expected dose	1 (1.8)	6 (11.3)	15 (30.6)	182 (73.1)	24 (34.7)

Patients with infused dose = expected dose	9 (15.8)	22 (41.5)	20 (40.8)	67 (26.9)	47 (65.3)

Patients with infused dose > expected dose	47 (82.5)	25 (47.2)	14 (28.6)	0	0

Results are expressed as number (percentage). Results are presented in patients with documented prothombin complex concentrate dose and initial international normalized ratio (INR) of at least 2 or unknown INR (*n *= 479).

**Table 5:** Vitamin K agonist resumption

	French guidelines			
	**Non-valvular atrial fibrillation**	**Venous thromboembolic disease**	**Mechanical heart valve**	

	**Should definitively stop taking VKA after ICH**	**Bridging anticoagulation period; VKA resumption to be discussed**	**Bridging anticoagulation period; eligible to resume long-term VKA**	

Overall population				Total

Number	342	101	38	516

VKA resumption	88 (25.7)	33 (32.7)	11 (28.9)	136 (26.4)

ICH				

Number	137	31	16	199

VKA resumption	15 (10.9)	4 (12.9)	4 (25.0)	21 (10.5)

Results are expressed as number (percentage). Results are presented in patients at day 15 after prothombin complex concentrate infusion. ICH, intracranial hemorrhage; VKA, vitamin K antagonist.

## Discussion

Here we report the characteristics and outcomes of patients with VKA-related bleeding and treated with PCC. Patients were recruited in emergency departments and intensive care units. To the best of our knowledge, this is the largest prospective 'real life' cohort reporting the management of patients with severe bleeding related to VKA.

In this study, severe bleeding sites were principally intracranial, and this is consistent with previously reported data. The most common risk factors for VKA-related intracranial hemorrhage are age and high-intensity anticoagulation [26,27]. As expected, the mean patient age in our study was 78 years, reflecting both the growing incidence of atrial fibrillation and the higher risk of VKA-related intracranial hemorrhage in older patients [28]. The mean patient age, the high-intensity anticoagulation (defined as an INR of greater than 3.5), the concomitant use of antiplatelets (in 22% of patients) [10], and the severity of bleeding may explain the large proportion of patients with intracranial hemorrhage.

Guidelines recommend PCC as the first choice or one of the first choices for VKA effect reversal [29]. PCC is able to normalize coagulation in a very short time, is often preferred to FFP, and is considered by some to be better tolerated [1]. Nevertheless, there are no prospective randomized controlled clinical trials comparing the various VKA reversal strategies. Vitamin K administration is essential in the coagulopathy reversal strategy but alone is not sufficient when rapid reversal is indicated [14]. Current strategies derived from consensus-based guidelines aim for rapid and safe coagulopathy correction [30,31]. Depending on whether INR is available, patients should receive either a dose adjusted to INR and body weight or a 25 IU/kg dose of factor IX. After PCC administration, INR should be carefully monitored to check whether the therapeutic objective (INR of less than 1.5) has been fulfilled. A second PCC administration may be necessary [23].

In emergencies, the initial INR is often unavailable before the administration of PCC. In our cohort, INR was unavailable for 86 patients (12.5%), although 300 (43.7%) presented with life-threatening intracranial hemorrhage. As the time frame between emergency admission and PCC administration was not collected, we cannot dismiss the possibility that PCC infusion had been delayed until the INR results were available, particularly in patients without hemodynamic instability. This could explain the low proportion of patients whose INR was unavailable. This time frame could also have an impact on prognosis. It is widely accepted that the shorter the delay for anticoagulation reversal, the better the prognosis [22].

INR monitoring in this study was clearly not in line with the recommendations. INR was first monitored with a mean time frame exceeding 4 hours after PCC administration, although INR monitoring is recommended at 30 minutes after infusion. Moreover, early INR monitoring enables us to administer further PCC doses when the target INR is not achieved. This critical stage in monitoring should be respected to improve INR normalization and hemorrhage control.

Target INR is clearly defined as 1.5 in the French guidelines. However, some authors claim that the therapeutic INR goal for intracranial hemorrhage should be not more than 1.4 and preferably not more than 1.2 [14,32-35]. The target INR was achieved in 452 patients (78.5%) in our study. This result is in accordance with the level observed in another series: 76.5% of patients with intracranial hemorrhage achieved an INR of less than 1.5 in a recent study by Desmettre and colleagues [36].

The proportion of patients reaching the target INR (< 1.5) was not impacted by initial INR (*P *= 0.18), although PCC was administered at lower doses than expected in the majority of patients with significantly increased INR (> 3.5). While PCC doses were expected to increase with initial INR, infused doses were similar regardless of the initial INR. Therefore, patients with an initial INR ranging from 2 to 3 received a higher mean dose than recommended, and 73.1% of patients with an INR of greater than 3.5 received a lower mean dose than recommended. PCC dosing thus was not always respected in view of the French guidelines. Patients received a mean PCC dose of 25 IU/kg, and initial INR did not seem to be considered in the PCC dosing. However, PCC dosing had no impact on the proportion of patients reaching the INR target or on the proportion of patients with bleeding control. In patients with intracranial hemorrhage, PCC dosing had no impact on survival, regardless of whether the recommendations were followed. Further studies are needed to assess whether adjusting the PCC dosage could improve survival and clinical outcomes.

It has also been suggested that doses of less than 500 IU could be appropriate if the INR is less than 5.0 [37]. This could explain why doses that were lower than expected resulted in similar outcomes. Furthermore, there is some evidence that an individualized dosage based on initial INR and body weight results in a greater proportion of patients who reach the target INR in comparison with the standard dosage [38]. Nevertheless, the question of whether to use an INR-based PCC dosage or a standardized fixed dosage remains open to discussion [26,39].

It is essential to include vitamin K in the treatment regimen since PCC has transient effects [40]. In our cohort, 576 patients (84.7%) received concomitant vitamin K. In a study including 18 patients with intracranial bleeding requiring neurosurgical intervention and treated with PCC (Kaskadil, LFB, Courtaboeuf, France). Vigue and colleagues [41] reported that INR was less than 1.5 within 6 to 12 hours after PCC infusion in 14 patients. The target INR was not reached in the four remaining patients for whom vitamin K had been omitted. In our cohort, no impact of vitamin K was found on target INR achievement.

Patients presenting with intracranial hemorrhage generally have a poor prognosis compared with that of patients with other bleeding sites. Survival rates were 75.4% in the overall population and 65.1% in patients with intracranial hemorrhage. VKA-related intracranial hemorrhages are reported to have a 50% mortality rate at 1 month [7]. The mortality rate of patients with intracranial hemorrhage depends of the site of hemorrhage [42,43], but unfortunately we did not collect details on the type of intracranial hemorrhage.

As highlighted by Makris and Van Veen [44] in an editorial introducing the results of a study by Imberti and colleagues [45], the fact that a significant proportion of patients died suggests that the PCC was appropriately administered to those with life-threatening bleeding. In our cohort, 52% of patients were older than 80, and old age is known to be a predictor of poor prognosis. Rapid anticoagulation reversal may lead to smaller hematoma and shorter delays before neurosurgical interventions and consequently to a better clinical outcome [46]. In our study, we found that achievement of target INR and bleeding control were strongly associated with survival. Antiplatelet agents have been suspected to increase hematoma growth and therefore to impair prognosis [47], but the mortality rate in this study did not differ, regardless of whether antiplatelets were used. The time between symptom onset and INR normalization is likely to be an important prognostic factor. Unfortunately, we did not study the time from symptom onset to admission or the time between admission and PCC administration, since our study was designed to describe the modalities of PCC use only. This time should be explored in a further study.

The anticoagulation reversal in patients with underlying thrombotic disease may be associated with a small risk of thromboembolic events. Given the heterogeneity in the composition of different formulations of PCC, the risk of thrombogenicity may vary [11,28]. The presence of procoagulant factors, such as factors II and X, and of preactivated factors, such as factor VII, increases the risk of thrombogenicity [48]. However, proteins C and S prevent excessive coagulation [49]. Risk factors are also related to the patient's underlying condition. Thromboembolic events could be due to a combination of several factors such as hypercorrection, hepatic dysfunction, and induction of a prothrombogenic activation. In our cohort, four out of 686 patients treated with PCC experienced thromboembolism: one presented ischemic stroke and three presented deep vein thrombosis. Stroke was considered by the physician to be possibly related to PCC. One further patient experienced DIC in a context of septic shock and no less than 21 days after PCC infusion. No patient experienced arterial thrombosis or heparin-induced thrombocytopenia. These results are in line with those of a meta-analysis (*n *= 460 patients) that reported a low thrombotic risk with PCC (1% to 5%) [50]. However, we acknowledge that thrombotic events could occur a long time after PCC infusion. In a study of 46 patients with intracranial bleeding, Imberti and colleagues [45] reported that two patients experienced thrombotic events 47 and 56 days after a three-factor PCC administration, although the authors concluded that these events were unlikely to be related to PCC administration.

Finally, depending on VKA indication, the French guidelines recommend stopping VKA or considering resumption. For patients with intracranial hemorrhage who have received VKA for non-valvular atrial fibrillation, VKA should be permanently stopped. However, VKA was resumed in approximately 11% of these patients in our study.

Our results have some limitations. We were interested in the modalities of Octaplex^® ^use and thus we did not collect data on other types of PCC. The rate of thrombotic events cannot be extrapolated to other types of PCC since there is a great disparity in PCC composition, particularly regarding proteins C and S and heparin. We cannot claim that our data are exhaustive, despite careful monitoring in each center. Some interesting data were not collected, including variables known to impair prognosis in intracranial hemorrhages, such as large hematoma, infratentorial hemorrhage location, low scores on the Glasgow Coma Scale, presence of intraventricular blood, QTc prolongation on an electrocardiogram, and high serum glucose at admission [7]. Patients were followed for 15 days, which is probably not long enough for older people and patients with intracranial bleeding. A better indicator of outcome would have been the outcome at hospital discharge. Finally, the patients in our study were identified from the list of patients with PCC prescription at the central pharmacy of each participating hospital. Therefore, patients with severe bleeding who did not receive PCC were neither identified nor studied.

## Conclusions

This large observational study showed that the management of severe bleeding related to VKA should be improved, particularly regarding PCC dosing, adequate INR monitoring, and systematic concomitant administration of vitamin K. The PCC was administered for severe bleeding related to VKA at a mean dose of 25 IU/kg, and INR normalization was achieved in the majority of patients. Bleeding control and target INR achievement were independent predictors of survival. Further studies are required to assess whether adjusting the PCC dose or better management of INR or both would improve survival rates.

## Key messages

• In the VKA effect reversal, a mean dose of 25 IU/kg PCC appears to be efficacious to achieve a target INR of 1.5.

• Target INR achievement and bleeding control are strong predictors for survival, regardless of hemorrhage site.

• In real-life conditions, management of severe bleeding should be improved with respect to PCC dosing and concomitant vitamin K administration.

• INR monitoring was not in line with French guidelines. The delay between PCC infusion and first INR monitoring should be shortened.

• Bleeding control and target INR achievement were independent predictors of survival.

## Abbreviations

DIC: disseminated intravascular coagulation; FFP: fresh frozen plasma; HR: hazard ratio; INR: international normalized ratio; PCC: prothombin concentrated complex; VKA: vitamin K antagonist.

## Competing interests

During the past five years, TD has received fees from Octapharma France (Paris, France) and LFB (Paris, France) for serving on advisory boards and providing lectures. C-MS has received fees from Octapharma, LFB, Baxter (Deerfield, IL, USA), and CSL Behring (King of Prussia, PA, USA) for serving on advisory boards and providing lectures. CG and CH have received fees from Octapharma France. PC works as an independent statistician and has received fees from Octapharma for conducting the study and analyzing data. JCC is an employee of Octapharma. RJ has received fees from Octapharma France, LFB, and CSL Behring. ED, FP, and SJ declare that they have no competing interests.

## Authors' contributions

TD participated in the design of the study and in the analysis of the results and wrote the manuscript. PC participated in the design and coordination of the study, performed the statistical analysis, and helped to draft the manuscript. JCC participated in the design and coordination of the study, reviewed the statistical results, and helped to draft the manuscript. All authors have read and approved the final manuscript.
